# Results from ^11^C-metformin-PET scans, tissue analysis and cellular drug-sensitivity assays questions the view that biguanides affects tumor respiration directly

**DOI:** 10.1038/s41598-017-10010-z

**Published:** 2017-08-25

**Authors:** Ane B. Iversen, Michael R. Horsman, Steen Jakobsen, Jonas B. Jensen, Christian Garm, Niels Jessen, Peter Breining, Jørgen Frøkiær, Morten Busk

**Affiliations:** 1 Department of Experimental Clinical Oncology, Noerrebrogade 44, Bldg. 5, Aarhus University Hospital, Aarhus, Denmark; 2 PET Centre, Noerrebrogade 44, Bldg. 10, Aarhus University Hospital, Aarhus, Denmark; 3 Institute for Molecular Biology and Genetics, Ole Worms Allé 3, Bldg. 1170, Aarhus University, Aarhus, Denmark; 4 Biochemical Pathology, Noerrebrogade 44, Aarhus University Hospital, Aarhus, Denmark; 5 Department of Endocrinology and Metabolism, Noerrebrogade 44, Aarhus University Hospital, Aarhus, Denmark

## Abstract

The anti-diabetic biguanide drugs metformin (METF) and phenformin (PHEN) may have anti-cancer effects. Biguanides suppress plasma growth factors, but nonetheless, the view that these mitochondrial inhibitors accumulate in tumor tissue to an extent that leads to severe energetic stress or alleviation of hypoxia-induced radioresistance is gaining ground. Our cell studies confirm that biguanides inhibits cell proliferation by targeting respiration, but only at highly suprapharmacological concentrations due to low drug retention. Biodistribution/PET studies of ^11^C-labeled metformin (^11^C-METF) revealed that plasma bioavailability remained well below concentrations with metabolic/anti-proliferative *in vitro* effects, following a high oral dose. Intraperitoneal administration resulted in higher drug concentrations, which affected metabolism in normal organs with high METF uptake (e.g., kidneys), but tumor drug retention peaked at low levels comparable to plasma levels and hypoxia was unaffected. Prolonged intraperitoneal treatment reduced tumor growth in two tumor models, however, the response did not reflect *in vitro* drug sensitivity, and tumor metabolism and hypoxia was unaffected. Our results do not support that direct inhibition of tumor cell respiration is responsible for reduced tumor growth, but future studies using ^11^C-METF-PET are warranted, preferably in neoplasia’s originating from tissue with high drug transport capacity, to investigate the controversial idea of direct targeting.

## Introduction

The biguanides metformin (METF) and phenformin (PHEN) are anti-diabetic drugs, but intriguingly, biguanides decrease cancer incidence and cancer-related mortality^1, 2^. Preclinical studies have demonstrated that biguanides inhibit cancer cell proliferation^3–5^, and may potentiate conventional treatment^4, 6–9^. METF has an excellent safety profile and is the first-line drug for managing type 2 diabetes, and exploring its significance in oncology is appealing. Accordingly, METF has moved into clinical testing, although the anticancer mechanisms remain unresolved^10^. The potent biguanide PHEN causes more frequent side effects, but its toxicity profile compares favorably with that of conventional chemotherapy and is under investigation as a cancer drug^11–13^.

Biguanides may principally impair tumor growth by indirect (systemic) or direct effects. Despite being both controversial and problematic, the idea that biguanides exerts direct effects in tumor tissue seems more appealing and has received immense attention^14, 15^. Biguanides are largely unable to enter cells by diffusion, and uptake relies on organic cation transporters (OCTs) which are abundant in liver (primary target organ), and kidneys^16^ whereas multidrug and toxin extrusion proteins (MATEs) are mainly involved in biguanide excretion. The working mechanism of biguanides is still debated, but it is generally accepted that biguanides work by mild inhibition of mitochondrial respiratory complex I in hepatocytes^17–19^, which triggers cell adaptive energy-saving measures, such as downregulation of macromolecule synthesis. In the liver, mitochondrial inhibition leads to a compensatory drop in glucose release^20^ which reduce plasma glucose and insulin levels, as well as insulin-like growth factors (IGFs) and cytokines. This may lead to a less favorable endocrine/metabolic environment for cancer development/growth and, importantly, endocrinological changes has also been observed in non-diabetic mice^21, 22^ and humans^23^. An indirect working mechanism was also proposed in a recent study, suggesting that the anti-cancer effect may be immune-mediated through a direct effect of metformin on specific immune cells^24^. Alternatively, biguanides may enter tumor cells causing direct metabolic effects, as proposed in numerous recent studies^25–28^. Due to high metabolic demands, an inefficient energy metabolism (Warburg effect) and a disorganized vasculature, tumors are characterized by low glucose and oxygen levels^29^. Adding further energetic stress by targeting of tumor respiration and/or glycolysis is, therefore, a promising strategy, which may slow tumor cell proliferation or even cause cytotoxicity in cells that are unable to compensate an ATP deficit or fine-tune ATP production and demand. Indeed, cells harboring mutations in liver kinase B1 (LKB1, upstream kinase of AMPK), which are quite common in some cancer types (lung, cervix), or cells with defects in the regulation of oxidative phosphorylation (OXPHOS) or glucose uptake are particularly sensitive to biguanides^25, 26^.

Evidently, the anti-cancer effects associated with biguanide treatment has catalysed intriguing and invaluable research that has identified metabolic weaknesses in certain cancers ability to handle energetic stress. However, basically all preclinical studies proposing that biguanides exert direct metabolic tumor effects have only observed *in vitro* effects at mM drug concentrations thus exceeding typical plasma levels in diabetes patients (<10 µM) 100–1,000 times^30^. Nonetheless, higher drug doses may be acceptable in an oncological setting and a sub-set of highly sensitive cancers where biguanides exert direct effects may exist, and if they do, identification of predictive biomarkers, such as genetic vulnerabilities or cellular drug retention, is highly warranted.

Since multiple OCTs and MATEs exist, drug retention may be difficult to predict based on gene expression studies, which may compromise their usability as predictive biomarkers^31^. Accordingly, we developed a method for labelling of METF with a positron emitter (^11^C-METF). This allows easy assessment of drug bioavailability *in vitro* and in tumor-bearing mice using different drug administration routes, with subsequent testing of pharmacologically relevant drug doses, using various metabolic/proliferative assays. Importantly, it also provides a clinical applicable tool to assess whether biguanides are retained in relevant quantities in human tumors.

## Materials and Methods

### Cells

A diverse selection of 16 cell lines, representing different disease and organ types and possessing distinct genetic/biological features (Supplementary, Table S1) that may affect drug retention and sensitivity, were included. Cells were cultured in MEM or HEPES-buffered Dulbecco’s modified Eagle’s medium (DMEM) supplemented with 10% FCS, non-essential amino acids, pyruvate and penicillin/streptomycin. To allow direct comparison, experiments were typically done in DMEM unless otherwise stated.

### *In vitro* glucose retention

The effect of biguanides on glucose metabolism was assessed from the retention of the ^18^F-labelled positron-emission tomography (PET) glucose analogue fluorodeoxyglucose (FDG). Cells were seeded on round cover slips in compartmentalized Petri dishes with free fluid movement between compartments^29^. Following attachment cells were flooded with DMEM. NCI-358 and LLC-PK1 adhere poorly to glass, and were seeded in plastic Petri dishes. When confluent, medium was renewed, and cells were exposed to the following aerobic conditions (21% O_2_/5% CO_2_): control, 500 µM METF, 50 µM PHEN or anoxic conditions (95% N_2_/5% CO_2_) with no drugs (anoxic control) or 50 µM PHEN administered in 100 µl saline. Anoxic conditions (<0.020 mmHg) was verified by inclusion of an anaerobic indicator strip. After 3 h treatment, FDG was added, and cells incubated for another 1 h. Cells were rinsed, and radioactivity for each cover slip was determined using a Packard well counter (Packard Instruments Co, Meriden, CT) and decay-corrected. Selected cell lines were also tested at higher/lower biguanide doses or under low glucose conditions (0.5 mM).

### *In vitro* metabolic characterization using a Seahorse Bioanalyzer

#### Acute biguanide treatment

oxygen consumption rate (OCR) and extracellular acidification rate (ECAR) were measured in eight cancer cell lines and a kidney epithelial cell line (LLC-PK1) using a Seahorse XF24 Extracellular Flux Bioanalyzer (Seahorse Bioscience). Cells were seeded at a density of 40,000–100,000 (cell-line dependent) in special 24-well plates. The next day, when cells had grown confluent, growth medium was replaced with pyruvate/glucose supplemented XF assay media. After a 30 min baseline measurement, drugs dissolved in incubation medium were added and changes in metabolism were monitored for 3 h.

#### Chronic biguanide treatment

To compensate for differences in growth rates and ensure appropriate cell number at the time of measurements, 5,000–20,000 cells were seeded per well. After overnight adherence, cells were treated for 96 h. To limit changes in culturing conditions, medium was changed once during this period. Finally, DMEM was replaced by XF assay medium containing the same drug concentrations, and metabolic fluxes were determined. Since cell numbers vary between the different treatments, results were expressed as relative glycolytic dependency (OCR/ECAR ratio).

### *In vitro* cell number/proliferation assay

A set-up was developed allowing quantification of a small number of cells grown in a large medium volume, thereby minimizing unwanted changes in medium composition. Cells (500 to 1,000) were seeded on cover slips placed in 12-well plates. When ready for experiments, cells were treated in specialized gas-tight chambers under standard or tumor microenvironment-mimicking conditions (22.5 glucose or 0.5 mM glucose under normoxia or anoxia). In a separate experiment, we also tested drug sensitivity under combined low glucose (0.5 mM) and low pyruvate (50 µM) in a normoxic atmosphere. Following 96 h of incubation/treatment, coverslips were fixed in methanol and stained with toluidine blue and digitalized (HP Scanjet 8300). Using ImageJ, images were binarized, and the stained area was expressed relative to control cells. Culture medium analysis revealed that glucose remained above 0.2–0.3 mM in the low-glucose groups.

### ^11^C-METF based assessment of *in vitro* cellular drug uptake

Cells were seeded in 5 cm plastic (NCI-358, LLC-PK1) or glass dishes and used for experiments at near-confluence. Cells were treated with vehicle (50 µl saline) or blocked with 10 mM unlabeled METF (in 50 µl saline), and tracer was added 15 min later. At appropriate time points, cells were washed thoroughly, and harvested by scraping and analyzed for radioactivity as above. In each experiment, a Petri dish was used for cell counting (hemacytometer). In five cell lines, we also assessed long-term uptake of METF. Due to the short half-life of ^11^C (20.95 min), ^3^H-labelled METF (PerkinElmer, Inc.) was used instead. ^3^H-labelled METF (92.5 KBq/dish) and 50 µM unlabeled metformin was added, and cells and medium samples were harvested 1, 2, 4 and 24 h after tracer administration, or 24 h after reincubation of tracer-loaded cells in tracer-free medium (to assess reversibility of drug retention) and cell number and radioactivity was determined (liquid scintillation counting). To allow calculation of cell-to-medium METF concentration ratios, average cell volume was determined in a separate series of experiments by simultaneous determination of cell number and packed cell volume using special centrifugation tubes (Techno Plastic Products AG, Trasadingen, Switzerland).

### Cellular transporter mRNA expression

Near-confluent cells were harvested in RLT buffer (Qiagen, Hilden, Germany) containing beta-mercaptoethanol. RNA was extracted using RNeasy columns (Qiagen), and cDNA was synthesized using random hexamer primers by the Verso cDNA Kit (Applied Biosystems, Life Technologies). All analyses were performed in duplicates using the “SYBR Green I Master” (Roche Life Science) in a LightCycler 480 (Roche Life Science). Relative gene expression was estimated by the default “advanced relative quantification” mode (software version LCS 480 1.5.0.39, Roche Applied Science). After assessing an array of reference genes GADPH (sense 5′-AATGAAGGGGTCATTGATGG-3′; antisense 5′-AAGGTGAAGGTCGGAGTCAA-3′) was considered superior. Target gene levels were expressed relative to the reference gene. The gene primer pairs are displayed in Supplementary Table S2. The PCR protocol was as follows: 10 s at 95 °C, 20 s at 60 °C and 10 s at 72 °C. The increase in fluorescence was measured in real time during the extension step.

### ^11^C-METF biodistribution and metabolic effects of treatment following different administration routes in non-tumor-bearing mice

Prior to experiments in immune-deficient tumor-bearing mice, we examined METF biodistribution and associated effects on glucose metabolism in conventional non-tumor-bearing CDF1 mice using two different routes of administration. To mimic a typical high-dose (yet well-tolerated) treatment protocol, trace amounts of ^11^C-METF were mixed with unlabeled METF in phosphate-buffered saline (PBS) equaling 250 mg/kg, and administered intraperitoneal (i.p.) or by oral gavage (per oral, p.o.). Tissues of interest were harvested 10, 30 and 60 min post-administration, weighed and analyzed for radioactivity as above and converted to equivalent tissue molarity. To assess metabolic consequences of biguanide treatment, mice were administered 250 mg/kg of METF or 100 mg/kg of PHEN i.p. or p.o. and administered FDG via the tail vein 1 h after treatment, and sacrificed after an additional 1 h. FDG uptake was quantified in various normal tissues as relative uptake by normalization to brain radioactivity, which is superior to conventional normalization to injected dose/weight^32^.

### ^11^C-METF *in vivo* biodistribution assessed by organ dissection and PET in tumor-bearing mice

NMRI-*nu/nu* athymic mice were whole-body irradiated with 4 Gy (to suppress any rest immunity) and inoculated subcutaneously in the back with A549 or SiHa tumor cells. To improve tumor take rate and growth, cells were mixed with a high concentration of Matrigel before inoculation^33^. When tumors reached ~500 mm^3^, mice were injected i.p. with a mixture of ^11^C-METF and unlabeled METF (250 mg/kg) and sacrificed 10, 30 or 60 min post-injection. Other, isoflurane anaesthetized, tumor-bearing animals were administered i.p. with a tracer/drug mixture (as above) or intravenous (i.v.) with a tracer bolus and PET scanned for 60–90 min followed by an anatomical magnetic resonance imaging (MRI) scan (for tissue delineation) using a Mediso nanoScan PET/MRI scanner. PET listmode data were reconstructed with a 3D iterative algorithm (Tera-Tomo 3D, full detector model and normal regularization; Mediso Ltd., Hungary) with four iterations and six subsets, and a voxel size of 0.4 × 0.4 × 0.4 mm. Data were corrected for randoms, dead-time and decay but not for attenuation and scatter due to the small size of the animals.

### Acute metabolic/microenvironmental effects in tumor and normal tissue in biguanide treated mice

Mice bearing SiHa or A549 tumors were administered vehicle (PBS), 250 mg/kg of METF, or 40 or 100 mg/kg of PHEN i.p. One h later, mice were administered FDG i.v. and the hypoxia marker pimonidazole i.p. (60 mg/kg). After another 1 h, mice were sacrificed and tissues and blood were collected, weighed and analyzed for radioactivity. Relative FDG uptake was quantified as ratios between different organs. Furthermore, 10 µm tumor cryosections were cut, dried and transferred to cassettes and overlaid with storage phosphor screens (SR2025, Fujifilm). After exposure, the FDG intratumoral distribution was read using a BAS5000 scanner (FUJIFILM, Japan). Tissue sections were subsequently immune-stained for pimonidazole-adducts and digitalized using a Hamamatsu Nanozoomer scanner and thresholded using ImageJ. In short, average background signal was determined in homogenous viable pimonidazole-free tissue areas and the threshold was set 50% above this background, resulting in maps displaying the distribution of viable hypoxic (oxygen partial pressure (pO_2_) <10 mmHg) cells and the hypoxic area fraction was calculated.

### Long-term PHEN treatment: effects on tumor growth, metabolism and hypoxia

Mice bearing SiHa or A549 back tumors were treated with 40 mg/kg PHEN or PBS i.p. twice per day (morning/afternoon, separated by ~8 h) when mean tumor volume reached 78 ± 6 mm^3^ and 66 ± 6 mm^3^, respectively. Tumor size was measured three times a week by caliper measurements. After 4 weeks, and 1 h after the last treatment, all mice were injected with FDG/pimonidazole and harvested and processed as described for acutely treated animals.

### Ethics approval

All animal experiments were conducted according to the animal welfare policy of Aarhus University (hppt://dyrefaciliteter.au.dk) with the Danish Animal Experiments Inspectorate’s approval 2012-15-2934-00087.

### Statistical analysis

Results are expressed as means (±SE). Statistical tests were performed as indicated in the figure legends and the significance level was set to p < 0.05.

## Results

### *In vitro* biguanide-driven changes in respiration and glucose consumption

**Figure 1:**
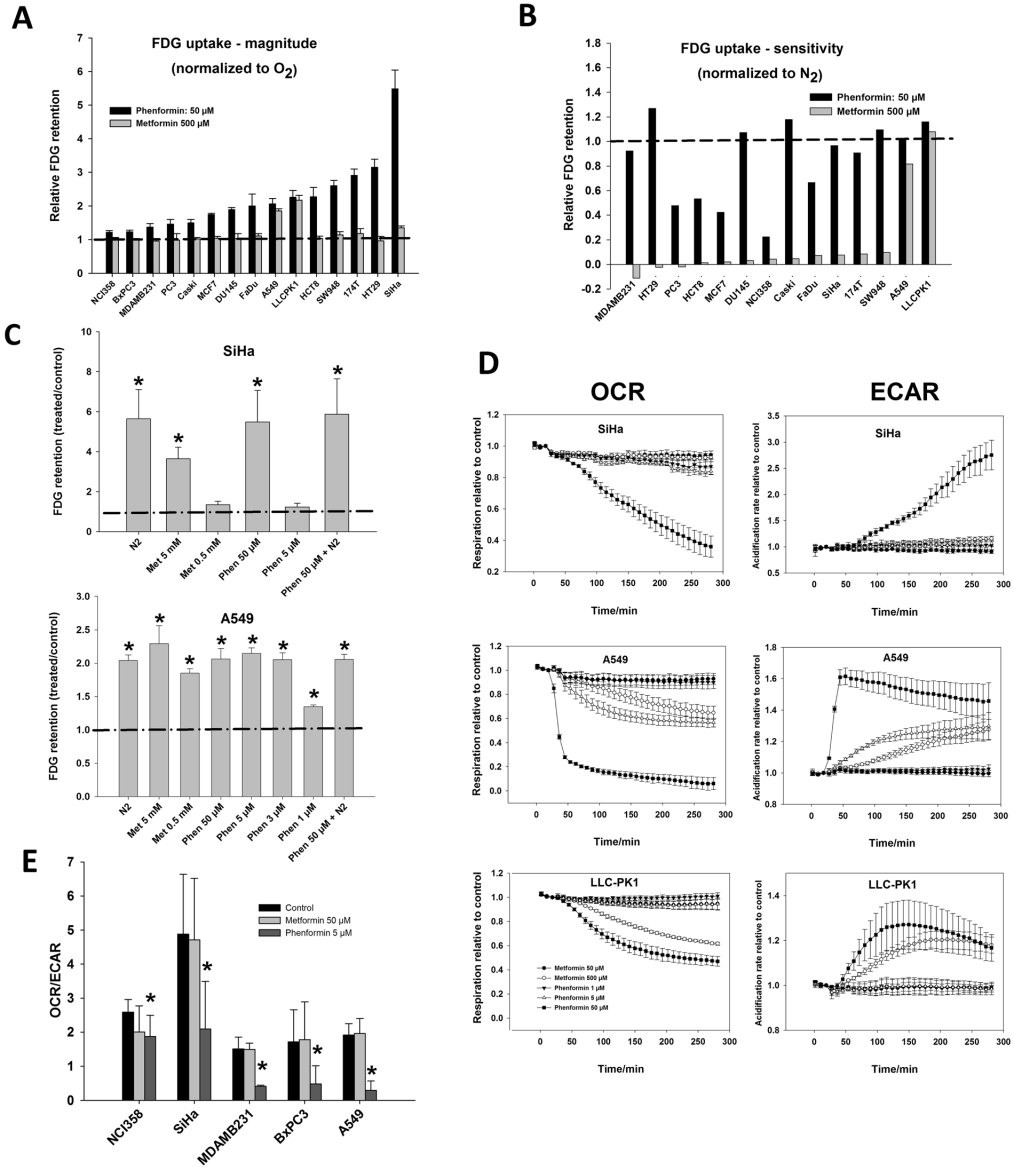
Metabolism in biguanide treated cells. Biguanide-induced changes in glucose metabolism in 14 cancer cell lines and a non-tumor kidney epithelial cell line (LLC-PK1). (**A**) change in FDG retention (relative to untreated cells) measured over a 60 min period starting 3 h after treatment initiation with 50 µM PHEN or 500 µM METF, where a value of “1” equals no change. Cells are ranked according to magnitude of maximal response. (**B**) treatment-induced change in FDG relative to change observed during complete mitochondrial inhibition (anoxic exposure, N_2_) calculated as: (FDG_treated_ − FDG_control_)/(FDG_N2_ − FDG_control_), where a value of “1” thus equals a change similar to that observed under N_2_ exposure and a value of “0” equals a value similar to oxygenated control cells. FDG uptake was not stimulated by N_2_ in BxPC3 and this cell line is thus not included in Fig. B. Cells are ranked according to sensitivity to METF. (**C**) changes in FDG retention (relative to untreated cells) in two selected cell lines when treated with a variety of concentrations of biguanides. For comparison, values observed during complete mitochondrial inhibition (N_2_) or a combination of N_2_ and 50 µM PHEN are also shown. (**D**) XFe24 Seahorse extracellular flux analyzer-based measurements of acute changes in cellular OCR and ECAR in cells treated with different concentrations of METF or PHEN. Results are expressed relative to untreated control cells. E: Flux analyzer measurements of energy metabolism following long-term exposure to lower levels of biguanides expressed as relative glycolytic dependency (OCR/ECAR) which is insensitive to treatment-related well-to-well differences in cell number. n = 3 or more independent experiments. A one-sample t*-*test was performed to assess if the relative fold-change in FDG uptake (normalized to their respective controls) was different from zero, whereas a two-tailed t-test was used to assess the significance of changes in the OCR/ECAR ratios (**E**).

Since biguanides are thought to work via mitochondrial inhibition, which stimulates glycolytic ATP production, we initially screened 15 tumor cell lines and a kidney epithelial cell line for changes in glucose consumption during drug treatment and complete respiratory inhibition (anoxia) using a FDG retention assay. Data revealed some very distinct outliers in terms of the maximum inducible metabolic flux changes and drug levels required to elicit such changes (Fig. 1A and B). Anoxia and high PHEN concentration resulted in a remarkably similar stimulation of glycolytic flux, and treatment under anoxia did not elicit any further increase in FDG uptake, suggesting that a biguanide-induced suppression of mitochondrial ATP synthesis drives glucose uptake (Fig. 1C and D). The kidney epithelial cell line LLC-PK1 and the NSCLC A549 were particularly sensitive but sensitivity was only observed at suprapharmacological drug concentrations. PHEN was typically ~100-fold more potent than METF (Fig. 1). The cervical squamous carcinoma cell line SiHa experienced by far the largest stimulation of FDG retention at a high PHEN concentration (Fig. 1A) and anoxia (Fig. 1C) but this is not due to a distinct drug sensitivity (Fig. 1B).

Based on the FDG assay, selected cell lines were further analyzed for changes in respiration and extracellular acidification rate (a surrogate marker for glycolytic flux) using a Seahorse XF24 flux analyzer. Seahorse data were in agreement with FDG data, but provides direct evidence that biguanides inhibit respiration at high doses (Figs 1D and S3 in supplementary). Differences in extra-mitochondrial O_2_ consumption may complicate inter-cell line comparisons of sensitivity, but addition of rotenone and antimycin A revealed that this was not the case (Supplementary, Fig. S1). Since biguanides may accumulate slowly, metabolic fluxes were also quantified in selected cell lines following a 96 h exposure period to low drug concentrations but all cell lines remained insensitive to pharmacological drug doses (Fig. 1E).

### *In vitro* cell-growth effects of biguanides at standard and tumor microenvironment-mimicking conditions

**Figure 2:**
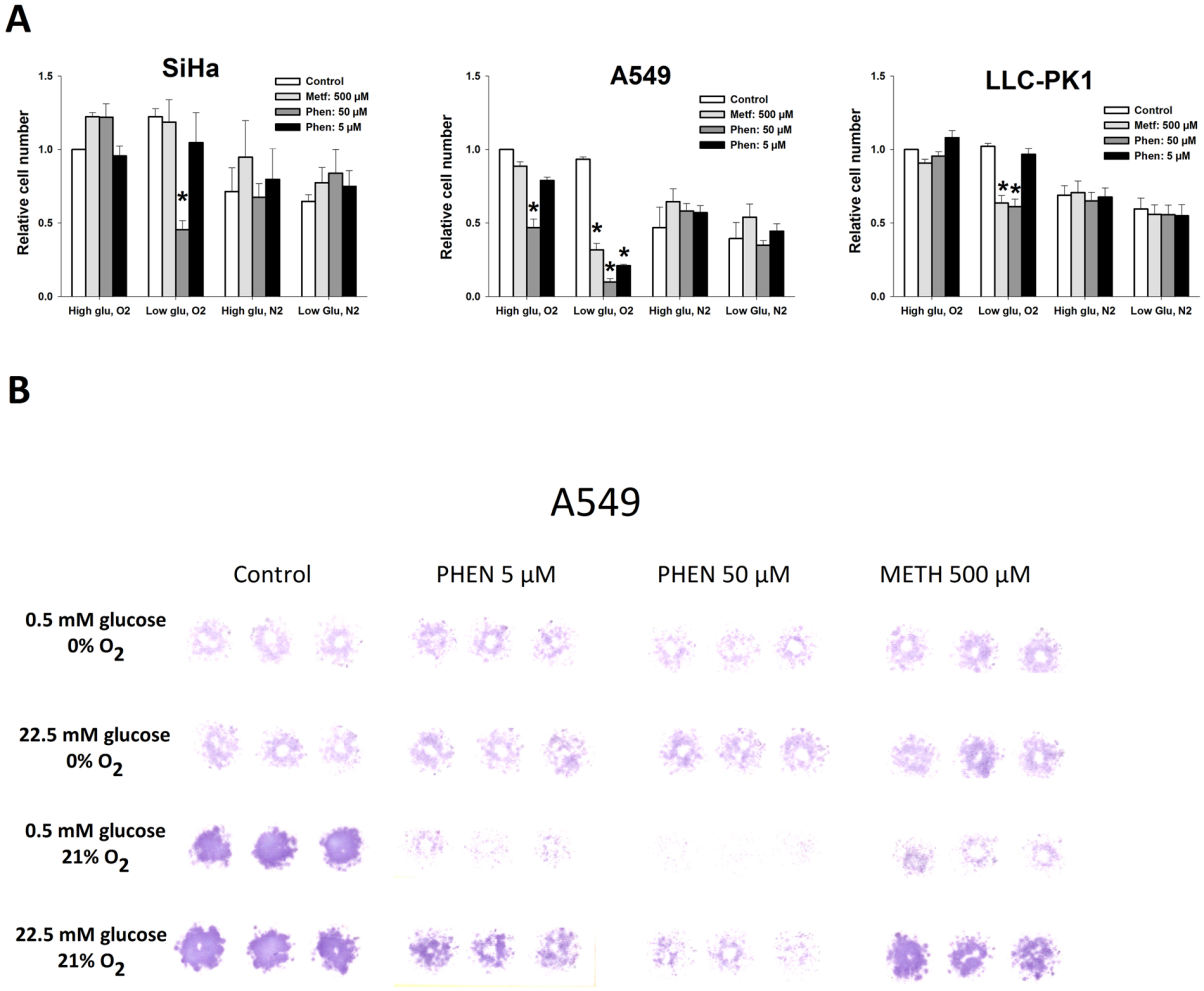
Cell growth-inhibitory effects of biguanides at various conditions that mimics the highly variable tumor microenvironment. (**A**) relative cell-number following 96 h treatment with biguanides under high (22.5 mM) and low-glucose (0.5 mM) glucose conditions in the presence (21%) or absence (0%) of oxygen quantified as stained cell area in treated versus untreated cells at control conditions (22.5 mM glucose and 21% oxygen). (**B**) a staining example of the highly sensitive A549 cell line. n = 3–4 independent experiments. A one-way ANOVA, followed by a Dunnet multiple comparison analysis was used to identify treatment groups differing from their respective controls.

This experiment was conducted to assess if metabolic effects translate into reduced cell growth rates and whether this was aggravated under energy-restricted conditions. Large differences between cell lines were evident, but in concordance with the metabolic assay, only highly suprapharmacological drug doses were effective. A549 and BxPC3 were particularly sensitive and low glucose (0.5 mM) augmented sensitivity (Figs 2 and S4) but combined exposure to low pyruvate (50 µM) and low glucose did not further sensitize cells (Supplementary Fig. S5). Anoxia reduced growth in most cell lines but interestingly anoxia fully eliminated the inhibitory effect of concurrent treatment. Of note, A549, MCF7 and SiHa performed better under anoxic conditions than during mitochondrial targeting under oxygenated conditions.

### Biguanide retention *in vitro*

**Figure 3:**
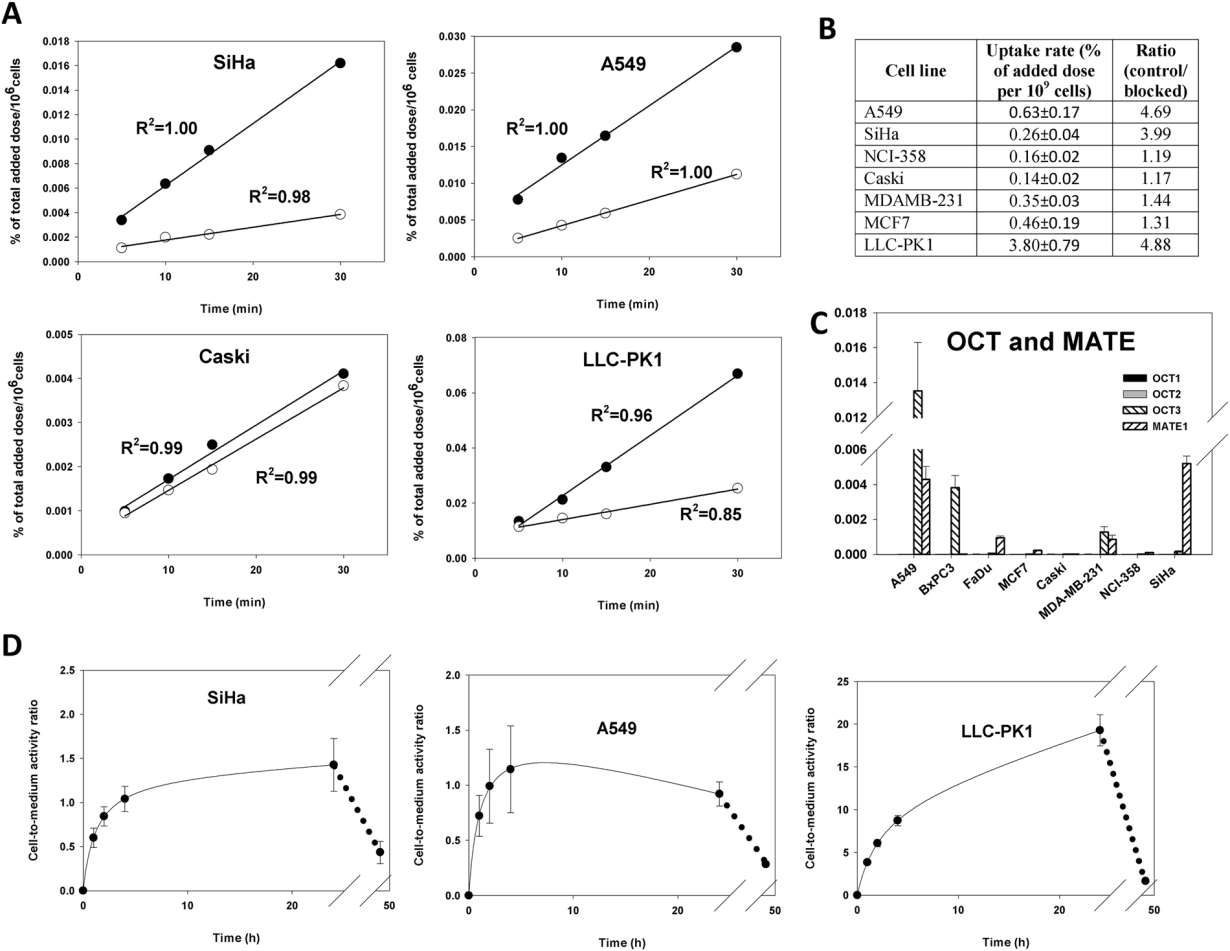
METF uptake and transporter expression in cells. (**A**) Short-term time-dependent cellular uptake of ^11^C METF for selected cell lines in the absence (filled symbols) or presence (open symbols) of 10 mM blocking unlabelled METF, expressed relative to total cell number. Uptake rates varied widely (notice that the scale on the Y-axis differs). (**B**) Uptake ratio between control and blocked cells. (**C**) OCT1, 2 and 3 and MATE1 mRNA levels expressed relative to the reference gene GAPDH in selected cell lines. (**D**) Long-term time-dependent uptake of ^3^H-labelled METF in the presence of 50 µM unlabeled metformin during a loading period of 24 h, followed by a 24 h period of incubation in tracer-free medium. Values are expressed as ratios between cell concentrations and medium concentrations. Notice the different scales on the y axes.

Next, ^11^C-METF was used to study correlations between drug retention and drug sensitivity *in vivo* (next section) and *in vitro*. LLC-PK1 displayed by far the highest uptake, whereas uptake in tumor cells varied widely being highest in A549 (Fig. 3). The effect of adding 10 mM unlabeled METF was mostly pronounced in cell types with high absolute uptake rates (LLC-PK1 and A549), suggesting that these cells rely on saturable transporter-mediated uptake (Fig. 3B and C). Since the short half-life of ^11^C prevents long-term studies, we used ^3^H labelled METF to assess tracer uptake and washout in LLC-PK1 and 4 tumor cell lines over 48 h (Figs 3D and S6) and expressed uptake as cellular concentration relative to medium drug concentration, taking into account differences in cell volumes (not shown). In tumor cells with transporter-mediated uptake (i.e., A549 and SiHa), cellular drug levels reached near-plateau values at 4 h or before. In Caski however, a plateau was not reached during the tracer incubation period and uptake was insensitive to 10 mM unlabeled metformin suggestive of slow diffusion-driven uptake (Figs 3D and S6). Notably, uptake in LLC-PK1 cells were 10 to 30 times higher than in tumor cells. Reincubation of drug-loaded cells in tracer-free medium revealed reversible drug retention in all cell lines (Fig. 3D).

### Bioavailability and bioaccumulation of METF and associated alterations in glucose use in mice

#### METF biodistribution and FDG use in non tumor-bearing mice

Tissue drug uptake depends on administration-route, plasma time-averaged drug concentration, tissue diffusion and *in vivo* transporter expression, and cell studies may not recapitulate *in vivo* tumor retention. Therefore, we initially assessed the influence of two METF administration routes on drug bioavailability/bioaccumulation (Supplementary Fig. S7) and on glucose metabolism (Supplementary Fig. S8), in non-tumor-bearing mice. A high METF dose of 250 mg/kg p.o. resulted in plasma concentrations of ~100 µM and considerably higher concentrations in liver and kidney. However, p.o. administration of METF (250 mg/kg) or PHEN (40 or 100 mg/kg) did not affect relative glucose (FDG) consumption, measured in the period from 60 to 120 min after treatment, even in organs with high drug uptake. Treatment with METF i.p. resulted in markedly higher concentrations in blood, liver and kidneys, and stimulated FDG retention in kidneys (both METF and PHEN) and heart (only PHEN) significantly, when normalized to brain (insignificant biguanide uptake).

#### METF biodistribution in tumor-bearing mice

**Figure 4:**
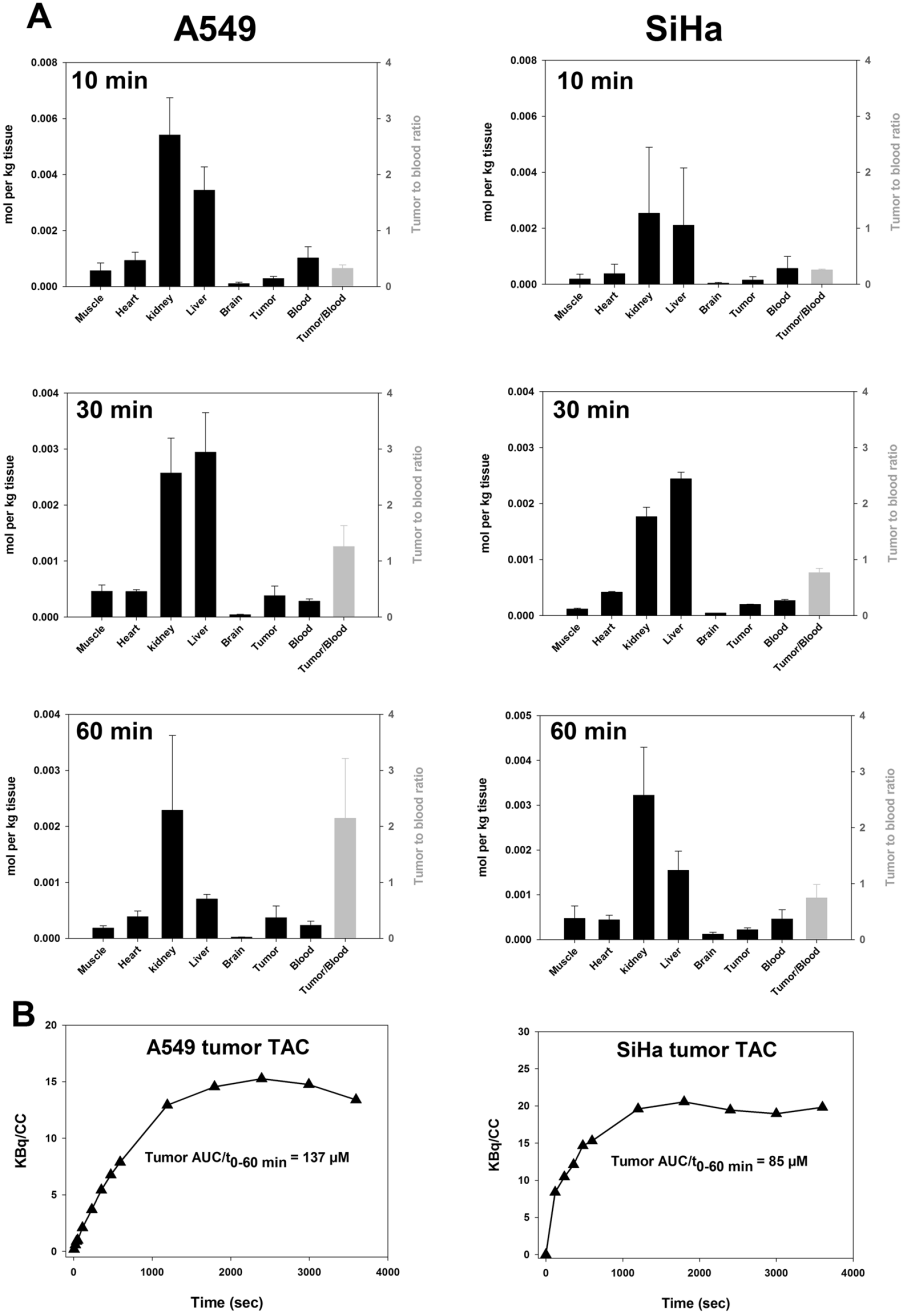
Biodistribution of ^11^C-METF in tumor-bearing mice obtained based on tissue dissection or non-invasively from PET-scans. (**A**) Mice were co-administered ^11^C-METF and 250 mg/kg of unlabelled METF (to simulate the treatment situation) i.p. and organs were harvested at different time points and radioactivity was converted to equivalent tissue concentrations. It is evident that most of the uptake is in the kidneys and liver, but some uptake was also observed in A549 tumor tissue with increasing tumor-to-blood ratios (secondary Y-axis) over time. n = 3 to 6 animals for each tumor model and sampling time point, except for SiHa 10 min where n = 2. Data are reported for whole-blood, but plasma levels may be up to two times higher if METF uptake by erythrocytes is insignificant. B: PET-scan derived raw time activity curves (TACs) for tumor tissue obtained from dynamic 60 min ^11^C-METF PET scans. Plotted radioactivity values are not corrected for differences in injected radioactivity dose, but from the TACs, average tumor METF concentrations over a period of 60 min was calculated (AUC/t_0–60_).

**Figure 5:**
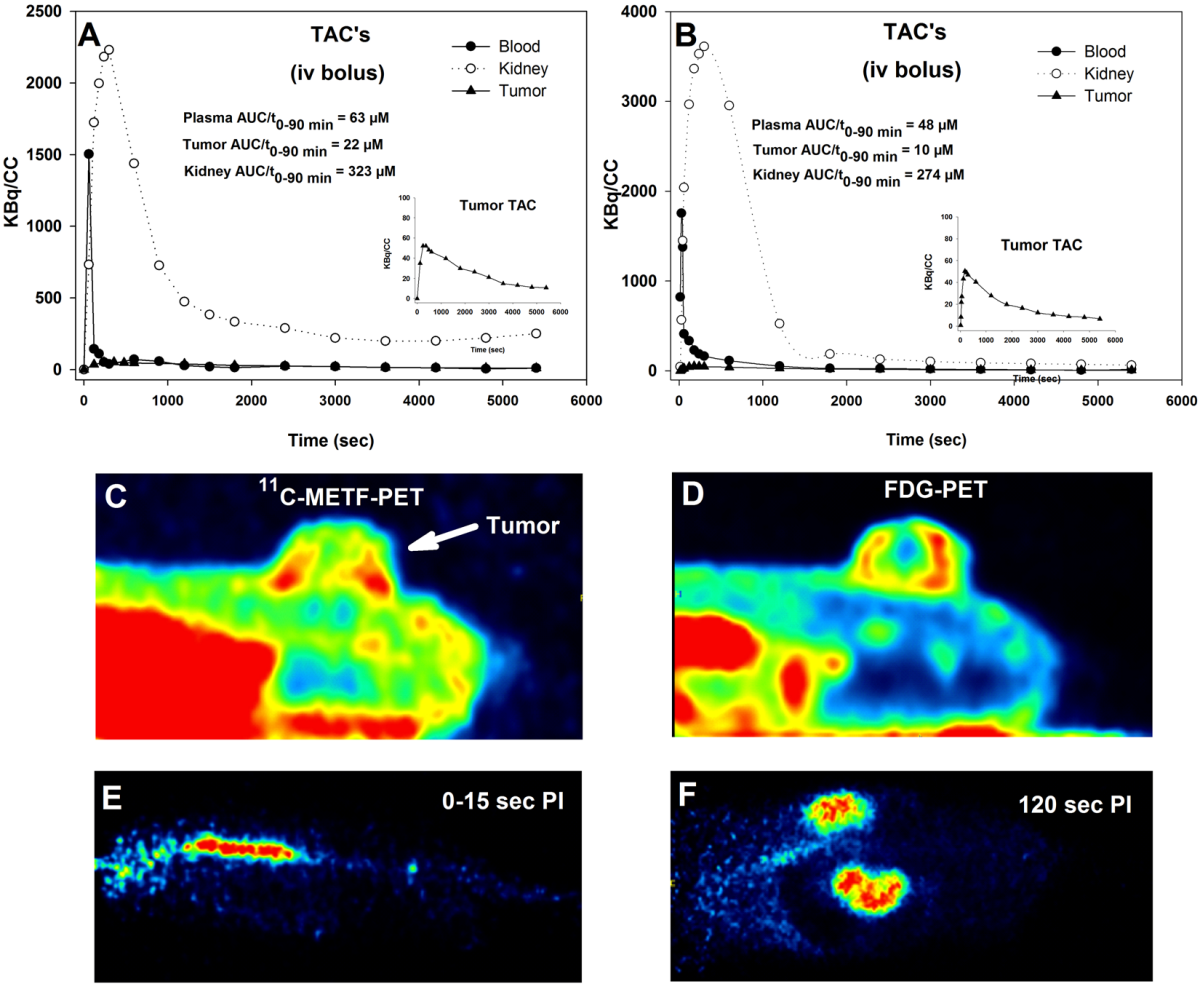
Dynamic ^11^C-METF PET-scans reveals fast drug dynamics and low tumor drug exposure. Time activity curves (TACs) showing radioactivity concentration over 90 min following i.v. injection of an ^11^C-METF tracer bolus were derived for organs of interest in a mouse bearing a SiHa (**A**) and a mouse bearing a A549 (**B**) subcutaneous tumor. (**C**) The A549 tumor was visible on late-time ^11^C-METF PET images despite much higher uptake in the abdominal area. (**D**) The same tumor following administration of the standard PET tracer FDG which revealed substantial central necrosis (see also Fig. 7) in this tumor model. (**E**) A blood volume was identified from the inferior vena cava during the first passage of the bolus, and the area under the curve (AUC) divided by time (mean radioactivity) was calculated which was finally converted to an equivalent expected mean drug concentration following administration of 50 mg/kg of METF. Mean plasma drug concentration was calculated from whole blood (image derived values) by assuming a haematocrit of 45% and no tracer leakage into erythrocytes and other blood cells. E: PET images showing the very fast distribution of METF with extensive tracer accumulation in the kidneys only 120 sec after tracer administration.

Since i.p. treatment resulted in higher bioavailability, this administration route was used for further experiments. Based on the characterization of *in vitro* drug sensitivity (Fig. 1B), metabolic responsiveness (Fig. 1A) and ^11^C-METF uptake (Fig. 3), A549 and SiHa cells were chosen as suitable *in vivo* tumor models. As in non-tumor-bearing mice, METF accumulated strongly and rapidly in liver and kidney (Fig. 4). Tumor retention was modest but tumor-to-blood ratio increased over time for both tumor models suggesting some tracer internalization and/or delayed washout from the interstitial fluid. Whereas the purpose of the previous experiment was to assess drug bioavailability during a high-dose treatment regime, i.v. administration of trace amounts of labelled drugs and PET-based compartment modelling of influx rate constants, may be very useful as a means to identify drug-retaining tumors in clinical situations. Accordingly, as proof of principle we also performed dynamic ^11^C-METF PET scans in i.v. administered tumor-bearing mice as summarized in Fig. 5. Our results revealed detectable, yet low, drug bioavailability (blood) and tumor retention, due to very fast drug clearance by the kidneys.

#### Normal tissue and tumor FDG retention and hypoxia

**Figure 6:**
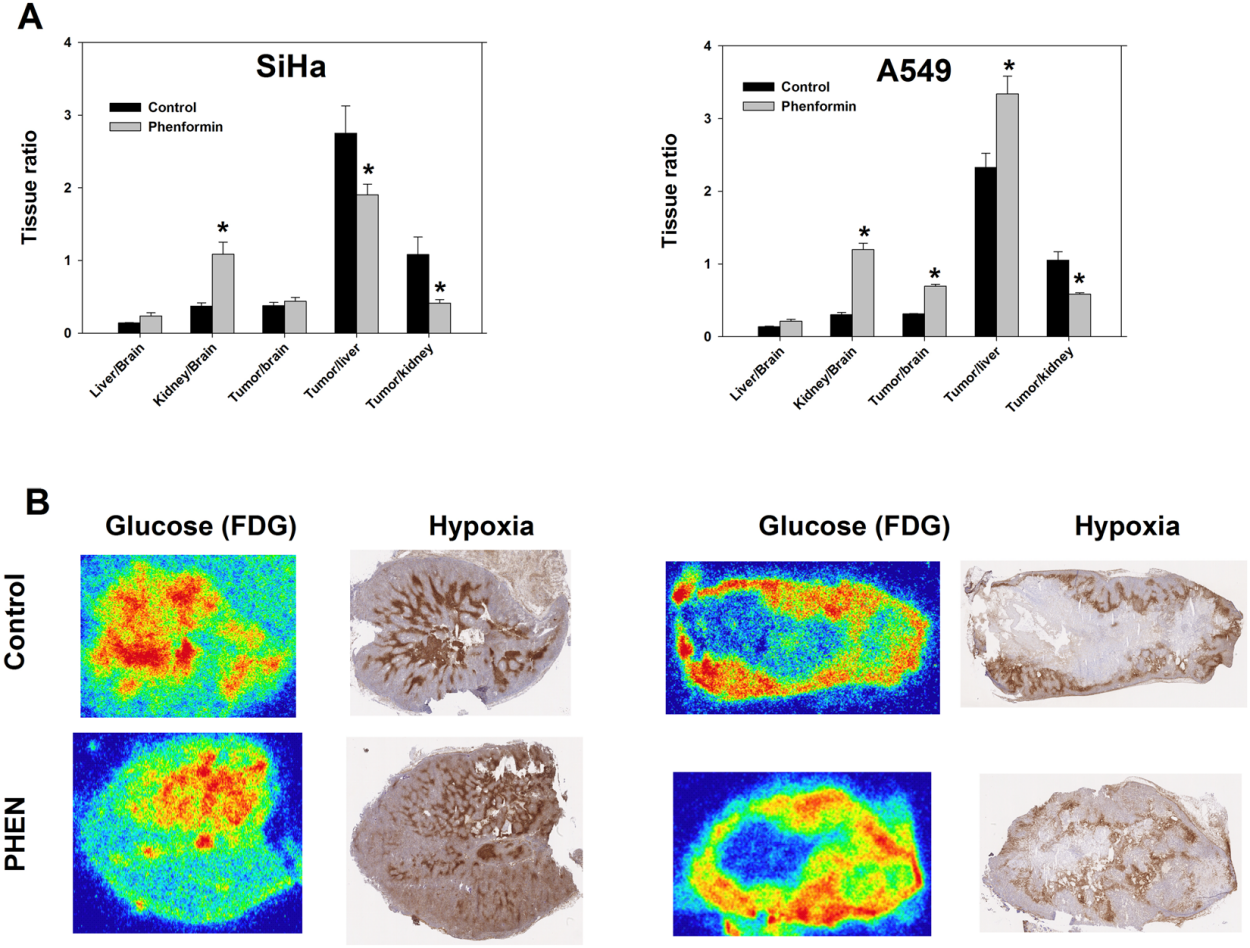
The effect of biguanide treatment on glucose metabolism and hypoxia in tumor and normal tissue. Tissue FDG distribution was assessed in tumor-bearing mice following i.p. administration of PBS or PHEN (100 mg/kg) dissolved in PBS. FDG was administered in the tail vein 1 h after treatment initiation, and was allowed to distribute for 1 h before tissue harvest. Bio distribution data are presented as tissue ratios between organs of interest using brain (negligible biguanide retention – Fig. 5) as a reference tissue to normalize against. FDG uptake was profoundly stimulated in kidneys but increased FDG retention was also observed in A549 tumors. B: Matching tissue slides showing glucose metabolism (FDG autoradiography) and hypoxia (pimonidazole) in tumor tissue cryosections. n = at least 3 animals. A two-tailed t-test was applied to assess the significance of changes in the various tissue ratios.

Based on our previous results we reasoned that PHEN was the biguanide of choice for further *in vivo* testing in tumor-bearing mice. Firstly, PHEN is ~100-fold more potent than METF *in vitro* (Figs 1 and 2) yet tolerated at a dose equaling 40% of that for METF *in vivo* and secondly, PHEN resulted in more pronounced acute changes in glucose metabolism in biguanide retaining tissues. As in non-tumor bearing mice, treatment stimulated kidney glucose metabolism very profoundly (p < 10^−5^ when using pooled data from all tumor-bearing mice) whereas liver uptake was slightly elevated (Fig. 6A). Treatment stimulated FDG retention significantly in A549 (p = 0.03) but not in SiHa tumors. Tumor hypoxia, quantified as hypoxic fraction (HF) based on the hypoxia probe pimonidazole, was not significantly affected by treatment (Fig. 6B). HF in viable tissue for the A549 tumor was 0.14 ± 0.01 and 0.16 ± 0.05 in PBS and PHEN treated animals, respectively, whereas HF for SiHa was 0.14 ± 0.03 and 0.07 ± 0.02 in PBS and PHEN treated animals. Necrotic fraction was ~50% in A549 whereas SiHa tumors contained little or no necrosis, but the extent of necrosis was unaffected by treatment.

### *In vivo* tumor growth inhibitory effects of PHEN treatment

**Figure 7:**
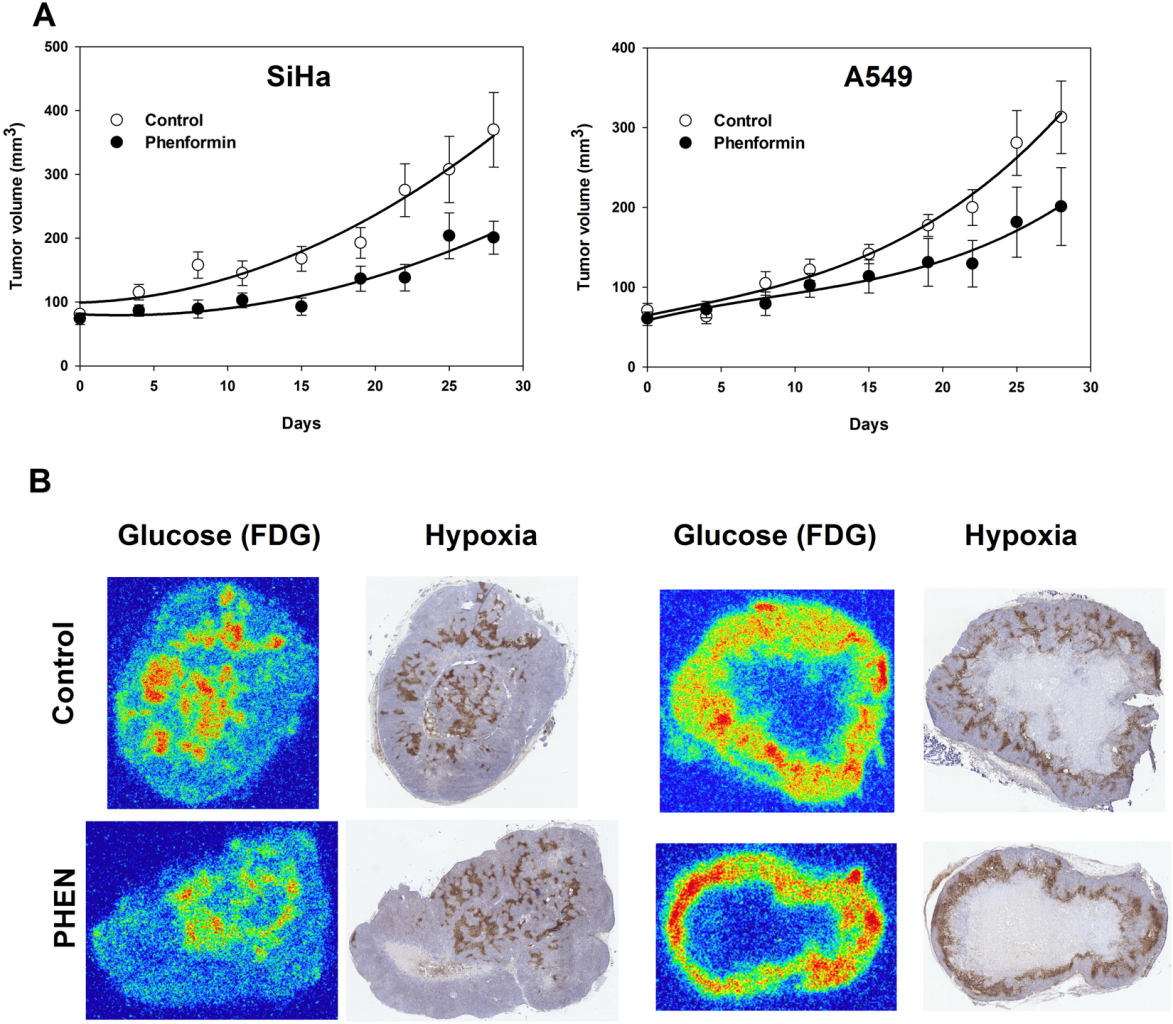
The effect of prolonged PHEN treatment on tumor growth, metabolism and microenvironment. (**A**) Tumor growth curves in size-matched tumors following treatment with PBS or PHEN (40 mg/kg i.p. twice per day) for 28 days. B: Matching tissue slides showing hypoxia (pimonidazole) and glucose metabolism (FDG autoradiography) in tumor tissue collected after the last treatment. n = 5–6. Growth inhibition was only significant in the SiHa tumor model as deduced from a two-tailed t-test comparing the relative area under the growth curve using the last four measurements.

Although mice tolerated single-dose PHEN treatment with 100 mg/kg well, unacceptable toxicity was observed during prolonged treatment. Therefore, tumor-bearing mice were treated with two daily i.p. injections of 40 mg/kg of PHEN (Fig. 7A). Treatment slowed tumor growth but the effect was not significant for A549 tumors. Reduced growth rates were not associated with changes in tumor glucose metabolism, hypoxia or the development of necrosis as quantified at the end of the treatment period. HF in viable tissue for the A549 tumor was 0.14 ± 0.01 and 0.16 ± 0.02 in PBS and PHEN treated animals, respectively, whereas HF for SiHa was 0.06 ± 0.02 and 0.04 ± 0.02 in PBS and PHEN treated animals. Necrotic fraction was not significantly influenced by chronic treatment and was 39 ± 6% and 47 ± 5% in A549 and 1 ± 1% and 0 ± 0% in SiHa following treatment with PBS and PHEN respectively.

## Discussion

The primary aim of this study was to develop a research tool that can advance our understanding of the anti-cancer working mechanism of biguanides, and potentially allow identification of patients that benefit from treatment. Specifically, we exploited our recent development of ^11^C-METF to allow assessment of biguanide drug retention in cells and *in vivo*. PHEN, which has received considerable interest in cancer treatment, has higher affinity for transporters and the mitochondrial membrane than METF. However, both compounds rely on the same transporters, and the carrier-mediated transport is much greater than transmembrane diffusion for either compound^34, 35^, suggesting that ^11^C-METF is suitable also for assessing tumor uptake capacity of related biguanides.

The anti-cancer working mechanism of biguanides is controversial but may involve indirect systemic effects mediated via changes in plasma hormones and metabolites resulting in a less favorable tumor microenvironment or direct, yet weak, effects on tumor cells that merely results in slowed tumor growth. However, several studies have proposed that biguanides, by targeting respiration directly, may acutely improve tumor oxygenation to an extent that sensitizes tumors to radiotherapy^8^ or induce cell death and tumor growth arrest as single treatment^25^ or when combined with other treatment to heighten energetic stress such as targeting of glycolytic energy production^27, 28^.

Our study support that biguanides *in vitro* at suprapharmacological concentrations, mainly works through inhibition of respiration whereas our data conflicts with a similar *in vivo* working mechanism. Specifically, we showed that: (1) anoxia and high biguanide concentrations elicited remarkably similar glycolytic flux increases and anoxia abolished any growth-inhibitory effect of METF/PHEN, supporting that respiration, *in vitro*, is a key target; (2) Energy metabolism and proliferation was unaffected by pharmacologically relevant concentrations, also under low-nutrient conditions which sensitize cells to mitochondrial inhibitors; (3) METF accumulated slowly in tumor cells compared to a kidney epithelial model cell and retention was reversible; (4) Biodistribution and PET scans revealed that i.p. injections increased drug bioavailability considerably compared to gavage, but tumor uptake plateaued ~45–60 min PI at much lower levels than in kidneys and liver, and tumor oxygenation was unaffected by high-dose biguanide treatment; (5) Chronic treatment reduced tumor growth in two tumor models but the response did not mirror *in vitro* drug sensitivity and tumor glucose metabolism and hypoxia was unaffected by treatment.

Tumor cell ^11^C-METF uptake varied widely but was much lower than in the kidney epithelial model cell line LLC-PK1 (Fig. 3). Generally, transporter-mediated (i.e., blockable) uptake (Fig. 3B) and OCT expression (Fig. 3C) were observed in metabolically (Fig. 1) and treatment (Fig. 2) sensitive cells and high METF sensitivity corroborated with high PHEN sensitivity. Of note, we observed distinct drug sensitivity in A549 and BxPC3, which has genetic defects that interferes with adaptive responses during energetic stress^25, 26^ (Table 1, Supplementary Material).

This suggests that ^11^C-METF-PET, possibly combined with genetic mutational analysis, could play a role in identifying patients with sensitive tumors. Importantly, however, METF doses exceeding typical plasma concentrations in patients or in mice treated via drinking water (<10 µM^36^ 50-fold or more, were required to elicit changes. A typical argument for explaining this profound discrepancy is that cellular drug retention and mitochondrial trapping may proceed during prolonged treatment, but no reasoning on why a similar mechanism should not be active *in vitro* has been given. Using ^3^H-METF, which allows prolonged drug exposure (Fig. 3D), we observed that in tumor cells with transporter-mediated drug uptake (Fig. 3) drug levels nearly plateaued at low levels close to medium concentrations within 2–4 h, whereas LLC-PK1 reached much higher levels. This corroborates well with the observation that 96 h treatment with clinical relevant drug concentrations were unable to affect energy metabolism (Fig. 1E) or slow proliferation even at low glucose/pyruvate levels (Figs 2 and S4 and S5), which has been shown to sensitize tumor cells to biguanides^34^. Extra-mitochondrial targets of metformin may exist (such as AMP deaminase or glycerophosphate dehydrogenase^35, 37^). Inhibition of glyconeogenesis is not likely to influence tumor growth and the observation that anoxia abolished the effects of treatment supports that the target during treatment with high drug doses is respiration. Our cellular studies shows that typical anti-diabetic doses are insufficient to affect tumor cell energy metabolism or proliferation *in vitro* but testing of higher doses may still be relevant. For example, it has been shown that OCT expression in freshly cultured hepatocytes drops rapidly over time^38^, which may justify the use of suprapharmacological *in vitro* doses to compensate for a loss in drug retention capacity. In addition, higher drug doses and alternative administration routes may be acceptable for treatment of cancer patients for a limited period.

We therefore, next studied the pharmacokinetics of metformin *in vivo* and compared normal and tumor tissue retention patterns, to changes in glucose metabolism and microenvironment. A high dose of 250 mg/kg (~15% of LD50) administered p.o. resulted in plasma METF concentrations up to 100 µM 10 to 60 min post-injection (Supplementary Fig. S7A), assuming insignificant METF uptake by erythrocytes, but did not affect glucose metabolism even in tissue with high OCT expression (Supplementary Fig. S8A). Administration by i.p. injection raised plasma concentrations to above 500 µM, whereas liver (main target organ) and kidney (main excretory organ) reached mM concentrations (Figs 4 and S7B). In accordance, kidney glucose consumption was markedly stimulated (Figs 6 and S8B), probably reflecting respiratory inhibition, although other explanations are possible. Unlike kidneys, FDG uptake was only stimulated modestly in the liver. However, since the liver supplies peripheral tissues with gluconeogenically-derived glucose and harbors glycogen stores, FDG may be a poor surrogate marker for inhibition of hepatocyte respiration, since energetic stress is compensated by reduced glucose release (probably why it works as a diabetes drug), rather than elevated uptake. Due to the short half-life of ^11^C biodistribution/PET studies are limited to a duration of ~90 min, but our data suggests that METF tumor uptake plateaued between 30 and 60 min post injection following a single i.p. dose (Fig. 4) at levels comparable to whole-blood levels (e.g. lower than plasma levels). Uptake was somewhat higher in A549 than in SiHa tumors (Fig. 4) but tumor retention was much lower than in liver and kidneys. This does not decisively exclude a direct effect on respiration in tumor tissue, since cell studies showed that A549, despite significantly lower drug uptake, was more responsive metabolically than LLC-PK1 (Fig. 1), especially towards PHEN, suggesting that biological differences modulates mitochondrial response at a given extra-mitochondrial drug load.

For further *in vivo* testing of metabolic and antitumor effects, we decided primarily to focus on PHEN, based on several observations. First, PHEN is 100-fold more potent than METF (Fig. 1). Second, METF had no *in vitro* effects (Figs 1 and 3) at *in vivo* achievable doses (Fig. 4). Third, PHEN was tolerated at a dose equaling 40% of METF and affected glucose use in organs with high biguanide uptake (Figs 6 and S8). Although i.p. administration resulted in much higher drug blood concentrations than reported in animals treated via their drinking water, invasive analysis of metabolism and hypoxia in tumor-bearing mice treated acutely with high doses of METF (not shown) or PHEN were inconsistent with direct effects on respiration. We did observe a borderline significant increase in FDG retention in the highly sensitive A549 tumor model (Fig. 6), but this was not a compensatory glycolytic stimulation caused by inhibition of tumor respiration, since tumor hypoxia was unaffected. Ultimately, we tested the influence of prolonged i.p. PHEN treatment in SiHa and A549 tumor-bearing mice. Surprisingly, the effect was slightly more pronounced (and only significant) in the relatively insensitive SiHa tumor which was unexpected if biguanides works directly in tumor tissue. In addition, there were no changes in tumor necrosis, glucose metabolism or hypoxia. Collectively, our data shows that metabolism can be affected selectively in organs with high drug retention, and that such effects potentially can be predicted based on pharmacokinetic studies using ^11^C-METF. However, our data does not support that biguanides, by direct inhibition of tumor respiration, can induce persistent energetic stress of a magnitude that is required for tumor growth inhibition or cell killing. Nonetheless, biguanides may still be useful as treatment sensitizers in certain tumors. Tumor hypoxia causes radioresistance but even a modest transient inhibition of tumor respiration may improve oxygenation significantly^39^. Indeed, Zannella *et al*.^8^ showed that administration of a bolus of METF (100 mg/kg i.v.) to tumor-bearing mice 30 min prior to radiotherapy improved treatment response, and concluded that METF inhibits tumor cell respiration directly. However, PET scans (Fig. 5) revealed that i.v.-administered ^11^C-METF clears very rapidly from the circulation, resulting in a predicted average METF plasma concentration of ~50 uM in the time interval from 0–30 min, even following administration of an extreme dose of 100 mg/kg (~60% of LD50^40^). Such low plasma concentrations are insufficient to elicit any respiratory response directly in tumor cells, even following 96 h exposure in monolayer cultures, without tissue diffusion barriers.

### Perspectives

A sub-set of cancers that are exposed to high drug concentrations due to a special location (e.g., urine-exposed superficial bladder cancer)^41^ or if they originate from tissues with high OCT expression (e.g., liver, kidney), assuming that high expression is maintained during oncogenesis, may exist. Such tumors are ideal for studies aimed at demonstrating a direct linkage between drug retention (^11^C-METF-PET) and changes in energy metabolism. It is mandatory that studies that claims the existence of a direct working mechanism based on observations of similar or overlapping cellular responses (e.g., AMPK activation) in cell studies using suprapharmacological drug doses and animal studies, further validate their reasoning for example by demonstrating that oct expression/drug retention differs widely between *in vitro* and *in vivo*.

## Electronic supplementary material

Supplementary Dataset 1
