# Supplementary material for: Results from 11C-metformin-PET scans, tissue analysis and cellular drug-sensitivity assays questions the view that biguanides affects tumor respiration directly
Source: Sci Rep. 2017 Aug 25;7:9436. doi: 10.1038/s41598-017-10010-z (PMC5573362; doi:10.1038/s41598-017-10010-z)
Supplement: Supplementary file 1 — Supplementary Dataset 1 [file 41598_2017_10010_MOESM1_ESM.doc]

Results from ^11^C-metformin-PET scans, tissue-analysis and drug-sensitivity assays questions the view that biguanides affect tumor respiration directly

**Ane B. Iversen, Michael R. Horsman, Steen Jakobsen, Jonas B. Jensen, Christian Garm, Niels Jessen, Peter Breining, Jørgen Frøkiær & Morten Busk**

**Supplementary material**

**Table 1.**

**Cell lines: origin and mutational status.** The table shows organ of origin and genetic/mutational status of genes that, when inactivated, may sensitize cells to biguanide treatment by compromising cell-adaptive responses to energetic stress, with special focus on LKB1 status, for all cell lines used in this study.

NSCLC: non-small cell lung carcinoma; SCC: squamous cell carcinoma; ADC: adenocarcinoma; LKB1: liver kinase B1

| **Cell line** | **Cancer/organ type** | **LKB1 status** | **Other** |
| --- | --- | --- | --- |
| **A549** | NSCLC | Truncated non-functional protein(1) | Unknown |
| **SiHa** | Cervix SCC | Gene deletion (2) | Unknown |
| **NCI358** | NSCLC | Wild type(1) | Unknown |
| **Caski** | Cervix SCC | Wild type(2) | Unknown |
| **MCF7** | Mammary ADC | Wild type(3) | Unknown |
| **MDA-MB-231** | Mammary ADC | No LKB1 mRNA(3) | Unknown |
| **PC3** | Prostate ADC | Unknown | Unknown |
| **DU145** | Prostate ADC | No LKB1 expression(4) | Unknown |
| **LNCAP** | Prostate ADC | Unknown | Unknown |
| **FaDu_DD_** | Head and neck SCC | Unknown | Unknown |
| **SW948** | Colon ADC | Unknown | Unknown |
| **HCT8** | Colon ADC | Unknown | Unknown |
| **HT29** | Colon ADC | Unknown | Unknown |
| **174T** | Colon ADC | Unknown | Unknown |
| **BxPC3** | Pancreatic ADC | Unknown | mtDNA encoded complex I mutation |
| **LLC-PK1 (non-tumor)** | Kidney, proximal tubule | Unknown | Unknown |

(1) Dahmani R, Just PA, Delay A, Canal F, Finzi L, Prip-Buus C, et al. A novel LKB1 isoform enhances AMPK metabolic activity and displays oncogenic properties. Oncogene 2015, 34:2337-2346. (2) Wingo SN, Gallardo TD, Akbay EA, Liang MC, Contreras CM, Boren T, et al. Somatic LKB1 mutations promote cervical cancer progression. PLoS One 2009; 4:e5137. (3) Shen Z, Wen XF, Lan F, Shen ZZ, Shao ZM. The tumor suppressor gene LKB1 is associated with prognosis in human breast carcinoma. Clin Cancer Res 2002, 8:2085-2090. (4) Yun H, Lee M, Kim SS, Ha J. Glucose deprivation increases mRNA stability of vascular endothelial growth factor through activation of AMP-activated protein kinase in DU145 prostate carcinoma. J Biol Chem 2005, 280:9963-9972.

**Table 2**

| h.SLC22A1/OCT1 | (sense 5’-TAATGGACCACATCGCTCAA-3’; antisense 5’-AGCCCCTGATAGAGCACAGA-3’) |
| --- | --- |
| h.SLC22A2/OCT2 | (sense 5’-ATGCCCACCACCGTGGACGAT-3’ ; antisense 5’-AGGAAGACGATGCCCACGTA-3’) |
| h.SLC22A3/OCT3 | (sense 5’-GGAGTTTCGCTCTGTTCAGG-3’; antisense 5’GGAATGTGGACTGCCAAGTT-3’) |
| h.SLC47A1/MATE1 | (sense 5’-TCGGCTTATCTTCTGCCTGT-3´; antisense 5’-CTGGGTAAGCCTGGACACAT-3’) |

Table 2. **OCT1, OCT2, OCT3 and MATE1 gene primer pairs.**

**Figure 1.**


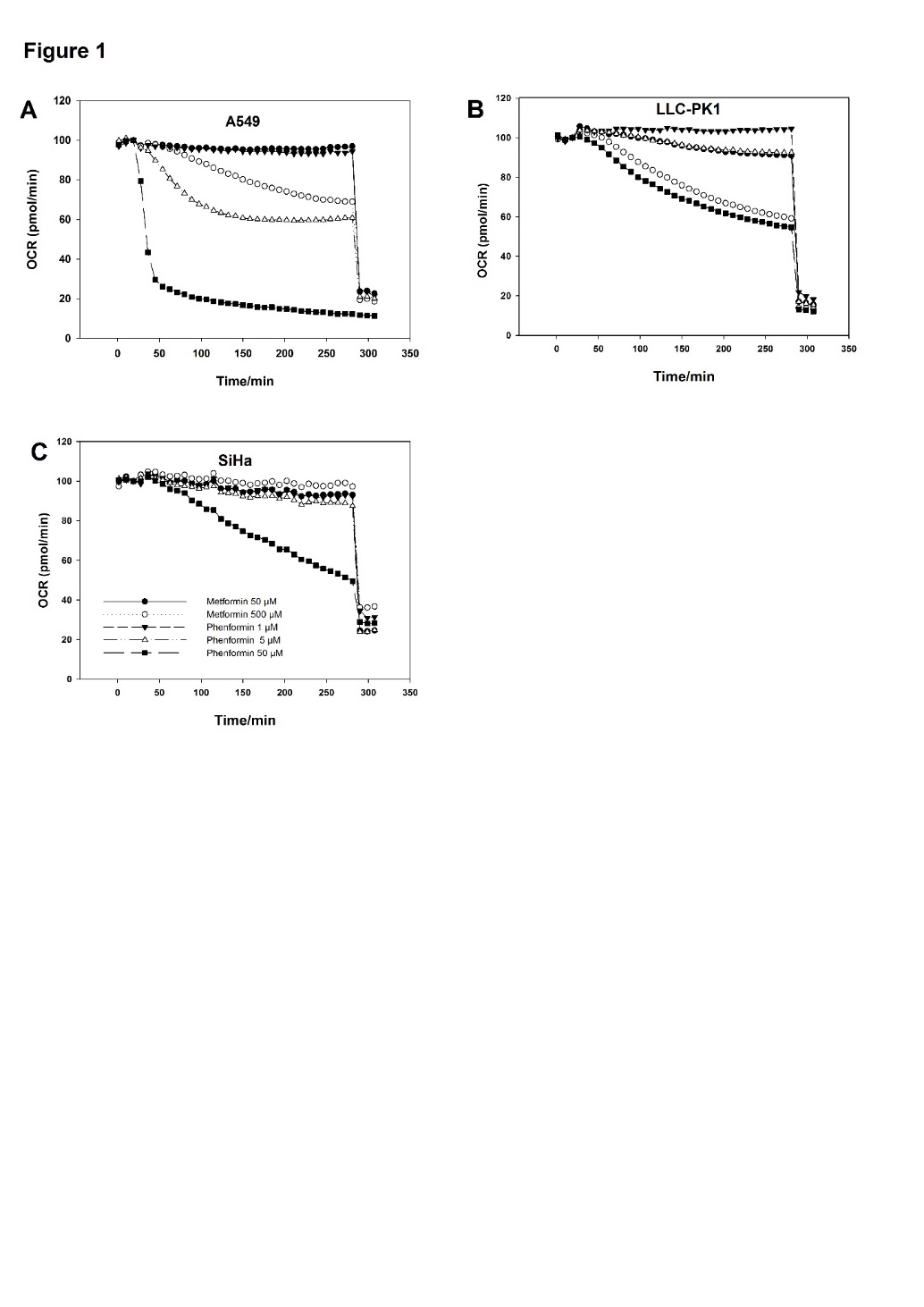

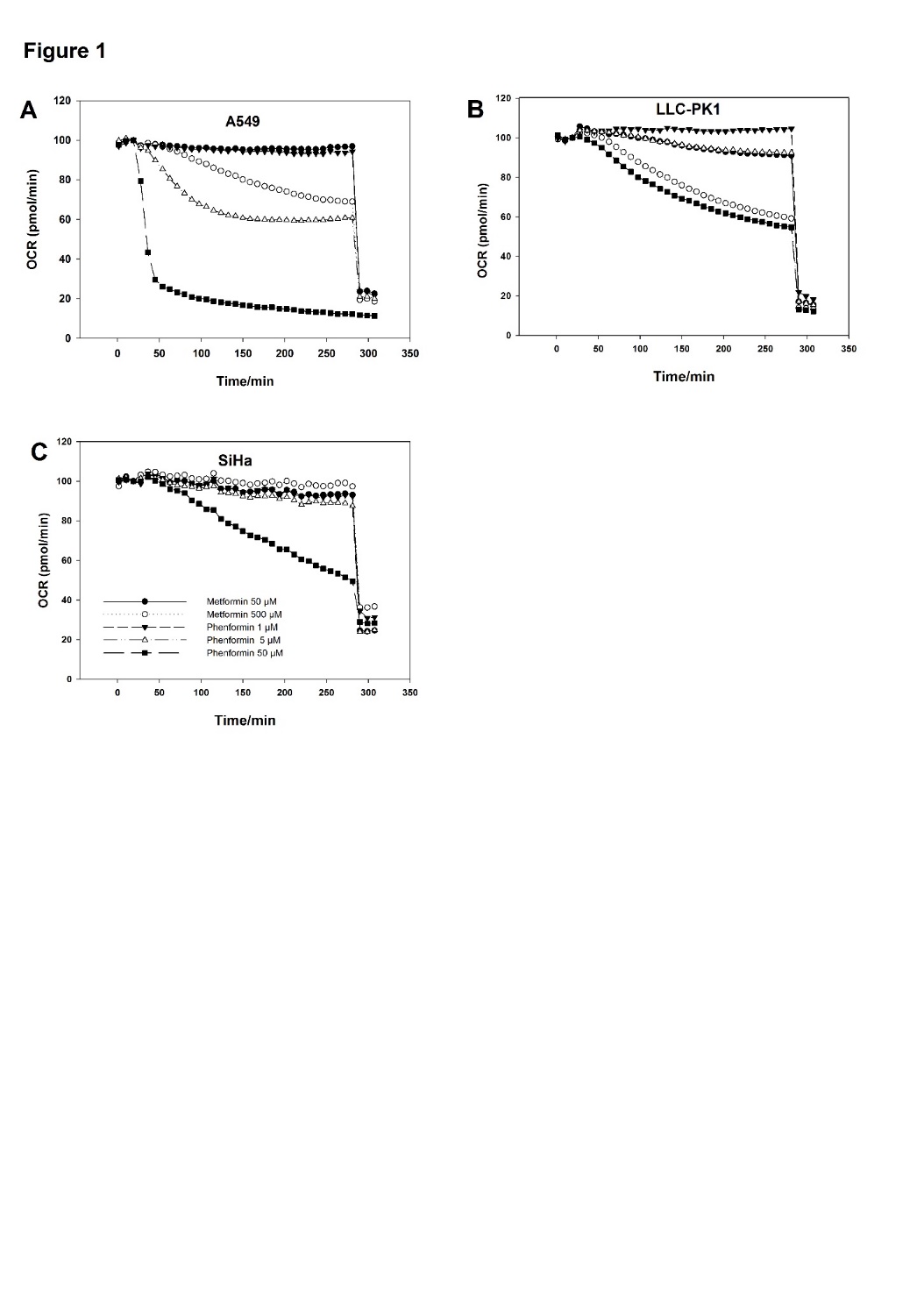


Figure 1. **Energy metabolism in selected cell lines treated with biguanides and subsequent rotenone/antimycin.** XFe24 Seahorse extracellular flux analyzer-based measurements of acute changes in cellular oxygen consumption rate (OCR) and extracellular acidification rate (ECAR) in different cell lines treated with different concentrations of MET or PHEN followed by treatment with mitochondrial drugs. Results are expressed relative to untreated control cells. It is evident that extra-mitochondrial oxygen consumption (remaining OCR following the addition of rotenone and antimycin) is low and rather similar in the different cell types.

**Figure 2.**

Figure 2. **Relative FDG retention in cells treated with biguanides at low glucose levels (0.5 mM)**. The anoxia/biguanide induced adaptive stimulation of glycolytic ATP production, as measured by FDG retention, was reduced in several cell lines compared to the response observed at 22.5 mM glucose (compare with figure 1A in the main manuscript), which may explain sensitization to mitochondrial inhibition during low-glucose conditions. Interestingly, low-glucose conditions resulted in a more pronounced response in MDA-MB-231, which may be linked to an acquired less glycolytic phenotype during low-glucose conditions. Results are means of at least three independent experiments. A one-sample t*-*test was performed to assess if the relative fold-change in FDG uptake (normalized to their respective controls) was different from zero.

**Figure 3**

Figure 3. **Changes in central energy metabolism in biguanide treated cells.** XFe24 Seahorse extracellular flux analyzer-based measurements of acute changes in cellular OCR and ECAR in a variety of tumor cells treated with different concentrations of METF or PHEN. Results are expressed relative to untreated control cells. Acidification rate is a surrogate marker for lactic acid production, which is typically elevated during inhibition of the respiratory chain to compensate the imposed ATP deficit. Results are means of at least three independent experiments.

**Figure 4.**

Figure 4. **Cell growth-inhibitory effects of biguanides at various conditions that mimics the highly variable tumor microenvironment.** Low glucose conditions sensitized some cell lines to treatment, but intriguingly, MDA-MB-231 was unaffected by low glucose and was stimulated by metformin treatment. BxPC3 was only tested for PHEN sensitivity. A one-way ANOVA, followed by a Dunnet multiple comparison analysis was used to identify treatment groups differing from their respective controls. Results are means of at least three independent experiments.

**Figure 5.**

Figure 5. **Relative cell number following 96h of treatment at low glucose and pyruvate levels to mimic the tumor microenvironment.** Cells were treated as stated in the figure, in medium containing 0.5 mM glucose and 50 µM pyruvate and cell number was normalized to untreated cells grown under similar low-nutrient conditions. A one-way ANOVA, followed by a Dunnet multiple comparison analysis was used to identify treatment groups differing from their respective controls. Results are means of three independent experiments.


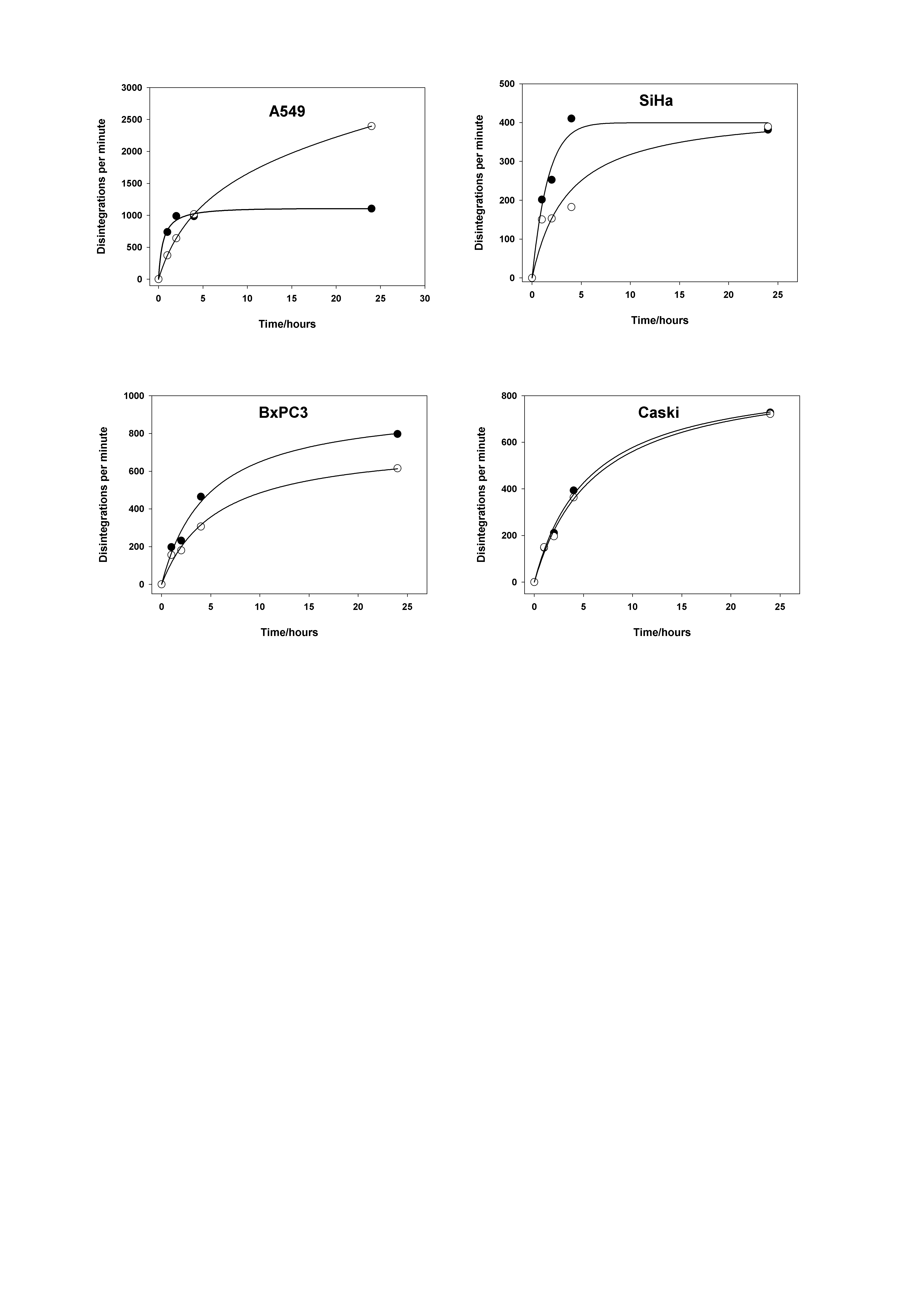
**Figure 6**

Figure 6. **^3^H-metformin retention in selected tumor cell lines measured over a period of 24h in the absence (filled symbols) and presence (open symbols) of 10 mM unlabeled metformin**. Uptake kinetics differs between unblocked and blocked conditions in cells with significant transporter expression (A549, SiHa and BxPC3), whereas there is no difference in Caski, which is characterized by low transporter expression levels (for expression data: see figure 3C in the main manuscript). A549 showed clear signs of cell damage and possibly compromised membrane integrity in the blocked group, which may explain the paradoxical uptake pattern with higher drug uptake in blocked cells at late time points. Results are means of at least three independent experiments.

**Figure 7.**


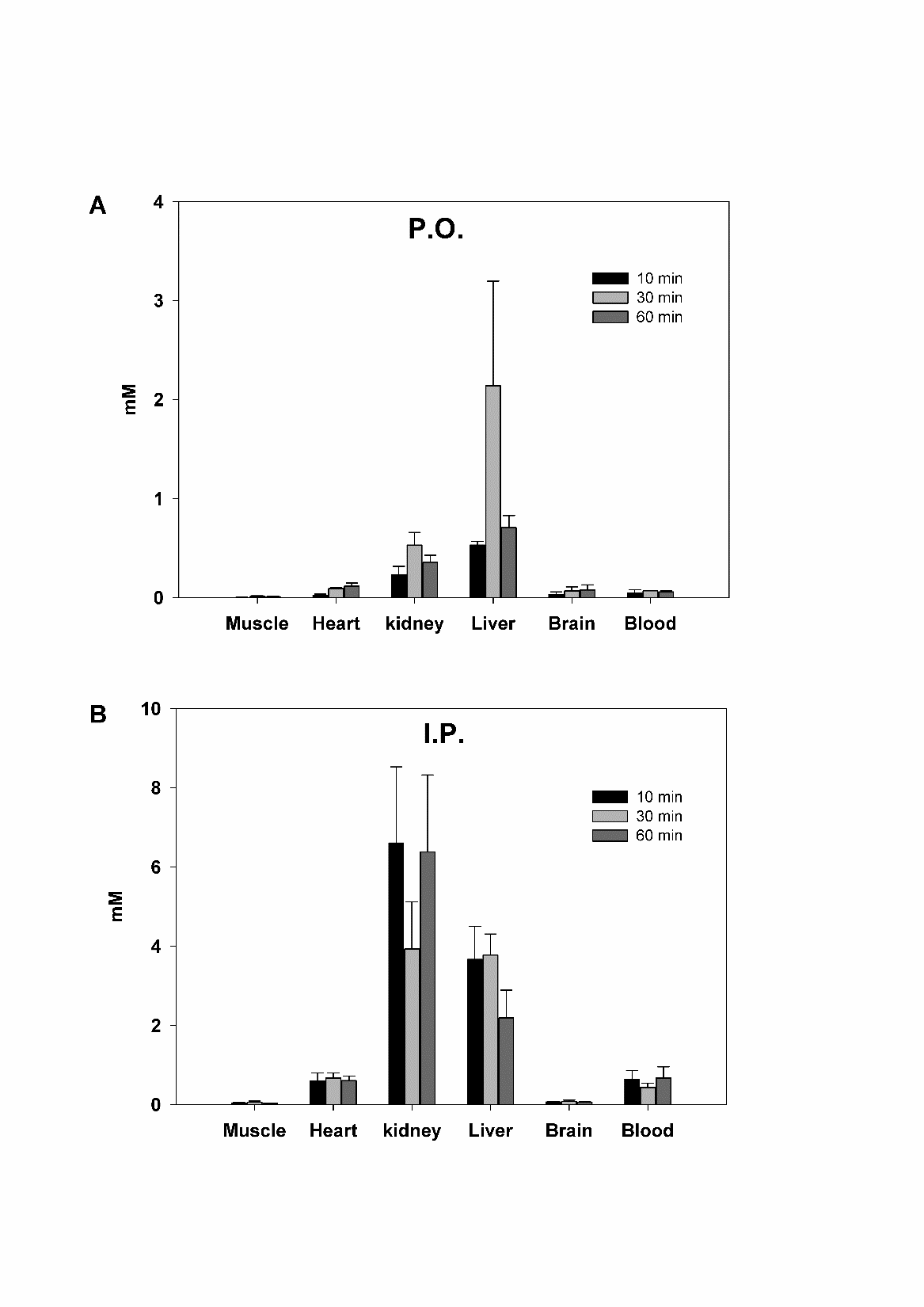


Figure 7. **Biodistribution of ^11^C-METF in non-tumor bearing mice.**  A: Mice were co-administered ^11^C-METF and 250 mg/kg of unlabeled METF P.O. (A) or I.P. (B) and organs were harvested at different time points (bar charts). Results are expressed as the mean (± SE). n=4-6 per group.

**Figure 8.**


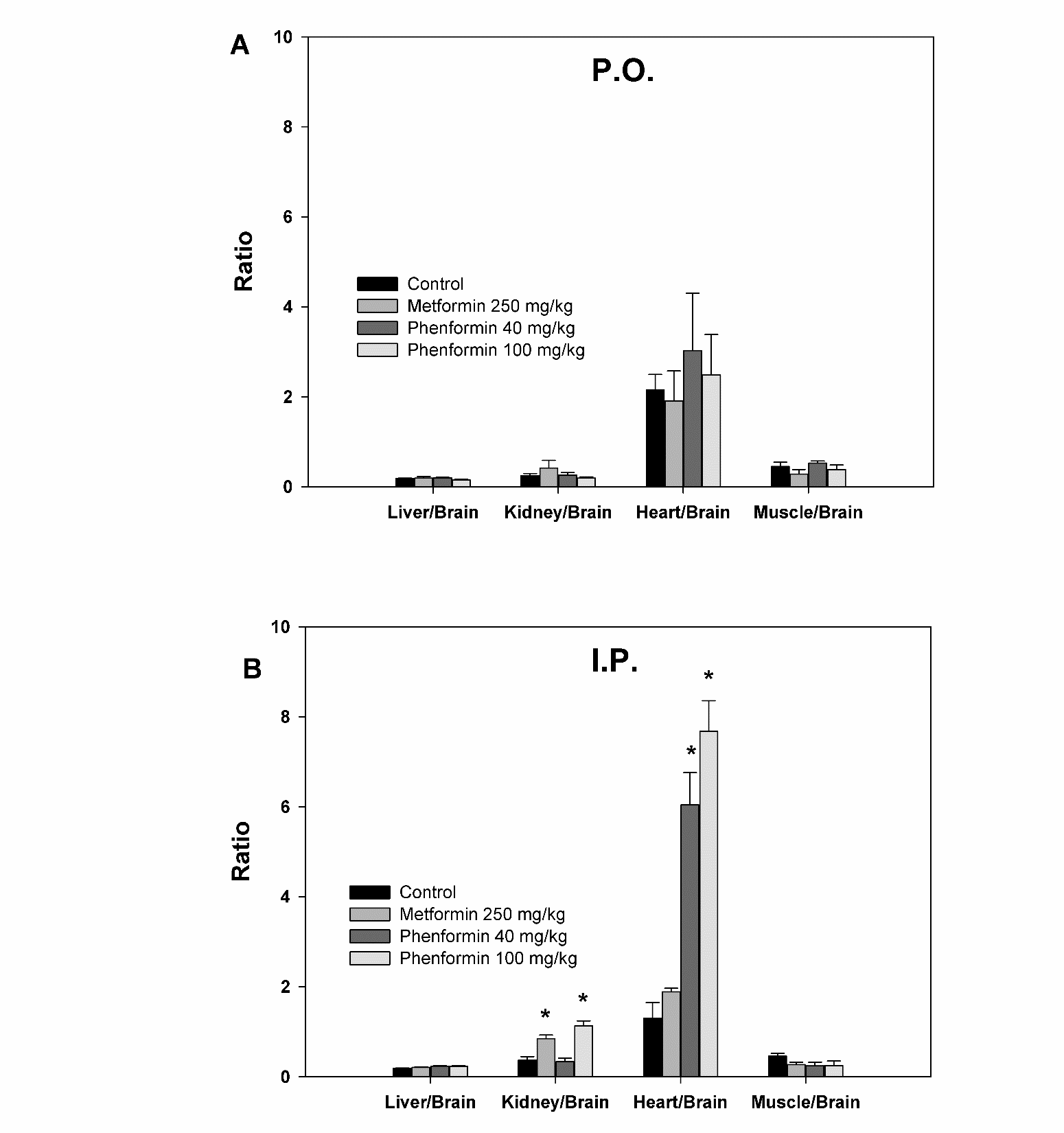


Figure 8. **Glucose metabolism in biguanide treated non-tumor bearing mice.** Biguanide-induced changes in FDG retention in mice treated P.O. (A) or I.P. (B). FDG retention is expressed as organ/brain ratios. Results are expressed as the mean (± SE) and statistical significant differences between measurements were determined using a one-way ANOVA. n=3-6 per group.
